# Re-thinking Physical Activity Programs for Older Brazilians and the Role of Public Health Centers: A Randomized Controlled Trial Using the RE-AIM Model

**DOI:** 10.3389/fpubh.2020.00048

**Published:** 2020-03-05

**Authors:** Tânia Rosane Bertoldo Benedetti, Cassiano Ricardo Rech, Lisandra Maria Konrad, Fabio Araujo Almeida, Fabiana A. Brito, Wojtek Chodzko-Zajko, Andiara Schwingel

**Affiliations:** ^1^Sports Center, Federal University of Santa Catarina, Florianópolis, Brazil; ^2^College of Public Health, University of Nebraska Medical Center, Omaha, NE, United States; ^3^Department of Kinesiology and Community Health, University of Illinois at Urbana-Champaign, Urbana, IL, United States

**Keywords:** physical activity, community, intervention, RE-AIM, public health center, behavior change, sedentary behavior

## Abstract

**Background:** Explored the role of public health centers in the delivery of physical activity programs to older Brazilians.

**Methods:** Total of 114 older adults (81% women) from public health centers across the city of Florianopolis, Brazil, were randomized into three groups: behavior change group (*n* = 36), traditional exercise group (*n* = 52), and control group (*n* = 26). The behavioral change group included 12 weekly meetings (2 h each). The traditional exercise group offered a 12-week exercise class. Individuals in the control group participated only in measurements. Program evaluation included a mixed-methods approach following the RE-AIM framework (reach, effectiveness, adoption, implementation, and maintenance). Trained interviewers conducted 12 focus groups and 32 interviews with participants in the program, professionals delivering the programs, community health workers, and local and city administrators overseeing public health centers. Participants completed health, quality of life, and fitness assessments at four time points.

**Results:** The study *reached* 11.5% of the eligible population in the community. Older adults' resistance to change and limited understanding of behavior change science by public health center staff hindered program reach. Physician encouraging patient participation and personal invitations by community health workers were perceived as favorable factors. Results of program *effectiveness and maintenance* suggest that behavior change strategies may be better suited than traditional exercise classes for decreasing sedentary time and increasing moderate-to-vigorous physical activity, as well as improving participants' quality of life. Only 14% of public health centers in the city adopted the programs. Heavy workload of health educators delivering the programs and limited physical space for program delivery were barriers for *adoption*. The fidelity of program delivery was high and indicates that the programs are culturally-appropriate for the Brazilian context and feasible for implementation by local health educators.

**Conclusions:** Our findings support the potential for dissemination of behavior change and traditional exercise programs to older adults through public health centers in Brazil.

**REBEC:** RBR-9pkxn2 (retrospectively registered) Register April 20, 2019.

## Background

Regular physical activity (PA) has been associated with maintenance and improvements in functional capacity and quality of life in older adults (1). Current guidelines suggest that older adults should strive to achieve 150 min/week of moderate-intensity aerobic activity, in addition to muscle-strengthening activities 2 days/week. Balance exercise is also recommended for many older adults as a way to prevent falls and fall-related injuries (2).

In Brazil, the 1990's were marked by an increase in physical activity promotion for older adults, when the Federal Government, States and Municipalities began subsidizing exercise classes to individuals for free or at low cost. The majority of these classes involved structured exercise programs (3) led by physical activity professionals or trained volunteers (4). In this article we refer to structured, instructor-led physical activity programs as “traditional” exercise programs. Common examples of traditional exercise programs include aerobics classes, aqua aerobics, team and individual sports, dance, and muscle-strengthening exercise. With an average of 30 older adult participants per class, these classes have the potential to help many older adults achieve the recommendation for PA, as they meet two to three times per week for an average of 60 min each time (4).

Despite the governmental efforts supporting the implementation of traditional exercise programs for older adults, PA participation remains disappointingly low across Brazil. For instance, the Brazilian national public health surveillance system (VIGITEL) assessments between 2011 and 2016 found no significant increases in leisure time PA, and reported that only 22% of adults 65 and older meet the recommendation for PA (5, 6). The surveys found that PA decreases with age and confirmed that Brazilian older adults are a vulnerable group for physical inactivity and related chronic diseases and conditions.

Research findings on the health impact of traditional exercise programs show that they are most effective when people participate regularly (7). Unfortunately, most older adults do not participate regularly in traditional exercise programs, which limits their effectiveness in increasing PA levels and providing health benefits. Additional limitations include low reach and high cost of these programs due to space, equipment and the need for instructor compensation (4). Although traditional exercise programs continue to be widely offered across Brazil, particularly at public universities, public health centers, and other public spaces, it is questionable whether or not this is a cost-effective strategy for PA promotion among older Brazilians (4).

In recent years significant attention has focused on the study of behavioral factors that increase the likelihood of an individual initiating and maintaining a regular program of exercise and PA. Research in this area suggests that incorporating a comprehensive behavioral management strategy in PA interventions can help maximize recruitment, increase motivation for exercise progression, and minimize attrition (8). Behavioral strategies include conversations about PA goals that are personally meaningful to individuals and help them find ways to make PA part of their lives (9).

The aging of society brings both opportunities and challenges for many low- and middle-income countries like Brazil. A new paradigm in health needs to be adopted, one that focuses on the prevention and management of chronic disease through healthy lifestyle strategies designed to maintain independent living and promote quality of life. In addressing this complex public health challenge, there is a need to bring community resources together and utilize systems that touch people's lives, including community and public health care settings. Public health centers, referred in this manuscript as HCs, are an example of a community health strategy sponsored by the government that has been Brazil's primary health care delivery strategy. Although most of the health services provided by HCs focuses on primary health care, prevention programs focusing on healthy lifestyles are increasingly being offered to communities.

In this article we describe our efforts to implement “Active Living Every Day,” an evidence-based program conceived and broadly disseminated in the United States, that incorporates behavior change for the promotion of PA (10). The goal of this study was to evaluate the potential of public community health centers for the delivery of traditional exercise classes and behavioral change programs for the promotion of PA among older Brazilians. This evaluation was guided by the RE-AIM framework (11, 12).

## Methods

Under the Unified Health System (SUS), cities across Brazil support teams of multidisciplinary professionals at local HCs to provide health care at no cost to about 4,000 people living in each neighborhood or community. Each team has a physician, nurse, technical nurse, and, at least, five community health workers with some also including nutritionists and exercise specialists.

**Figure 1:**
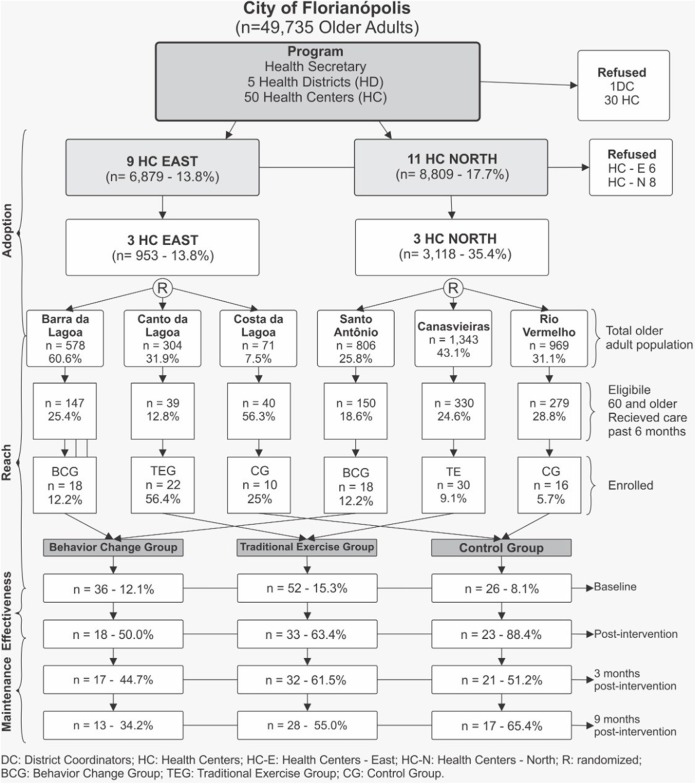
Flow diagram of study participants in an intervention of health centers (HC). Florianopolis, Brazil, 2012.

This study took place in Florianopolis, Santa Catarina, a city with ~500,000 inhabitants in Southern Brazil. In Florianopolis there are 50 *Public Health Centers (HC)* divided in five health districts. The implementation of our project started with an initial meeting where the study was presented to all five health district coordinators. Of those, two coordinators (districts North and East) demonstrated interest in participating in the study. A total of 20 HCs belonged to districts North (*n* = 11) and East (*n* = 9), but not all of them had the physical structure and human resources to offer the programs. Thus, a total of six HCs were involved in the study and randomized to one of three groups (Traditional Exercise Group—TEG, Behavioral Change Group—BCG, Wait List Control Group – CG) stratified by health district (see Figure 1).

## Participants

Study participants were men and women aged 60 or older who had no severe physical and/or mental health impairments and had not participated in physical activity programs in the past 6 months. Exclusion criteria included: history of heart attack and/or stroke in the past 6 months, cancer diagnosis and/or other severe medical conditions. Strategies to recruit participants included local media advertisements, flyer distribution, referrals by HC team members during medical appointments and home-visits by community health workers.

A total of 114 older adults were enrolled and assigned to one of three groups based on their home HC assignment: traditional exercise, behavior change, or waiting list control group. After initial group assignment, an orientation session to explain the study was offered to all interested older adults. Those interested in joining the program, completed the enrollment process, signed the informed consent and scheduled the appointment for baseline data collection. The protocols were approved by the ethics committee of the Federal University of Santa Catarina (CONEP n. 480560 and CEPSH n. 2387/2010).

## Groups and Program Description

### Behavior Change Group (BCG)

The BCG participated in a behavioral change program that was adapted from “Active Living Every Day,” or ALED (13). ALED is an evidence-based program conceived and broadly disseminated in the United States that assists individuals to become more physically active. ALED is structured into 12 weekly meetings of 1.5–2 h duration. The sessions follow a series of topics related to behavior change with the goal of achieving a more active lifestyle (Table 1). Meetings were conducted by nutrition or exercise science professionals already working at the HCs who received specific training to facilitate the program. An agreement was established with Human Kinetics®, copyright holder of ALED, for using the program in Brazil. It included training of program personnel, rights to adapt/translate ALED into Brazilian Portuguese. ALED was linguistically and culturally adapted by Benedetti et al. (4).

**Table 1:** Chapters of the Behavior Change Program—VAMOS—Brazil, 2012.

**Active living every day program**	
Week 1. Ready, Set, Go.	Week 7. Avoiding pitfalls.
Week 2. Finding new opportunities.	Week 8. Step by step.
Week 3. Overcoming challenges.	Week 9. Defusing stress.
Week 4. Setting goals and rewarding yourself.	Week 10. Finding new ways to be active.
Week 5. Gaining confidence.	Week 11. Positive planning.
Week 6. Enlisting support	Week 12. Making lasting changes.

### Traditional Exercise Group (TEG)

Participants in the TEG received a 12-week exercise class conducted at the local HCs. The classes were held three times per week for 60 min duration. The classes included a 5- to 10-min warm-up, 25 min of aerobic exercise at 50–80% of max. aerobic power, resistance training for 20 min, and a 5-min cool-down. Participants had their heart rate and ratings of perceived effort tracked throughout the sessions (14). Classes were led by exercise professionals employed by the HC.

### Wait List Control Group (CG)

Individuals in the CG participated only in measurements, without any intervention. They were asked to continue their routine activities before the start of the study. At the completion of the 9-month post-randomization assessment they were offered participation in the TEG classes.

## Program Evaluation Using the RE-AIM Framework

We chose to conduct a comprehensive program evaluation including all dimensions of RE-AIM (11) using quantitative and qualitative data. Accordingly, we assessed reach (participation rate and representativeness), effectiveness (impact on health outcomes), adoption (interest in the program), implementation (consistency of delivery and costs), and maintenance (impact on long-term outcomes, continuing to offer the intervention over time). Our mixed-methods approach builds on successes of prior studies that have focused on program evaluation (15, 16). Trained research personnel conducted a total of 12 focus groups (FGs) and 32 interviews including: the director of the City Health Department, managers and the coordinators of HC/NASF Family Health Support Centers, coordinators of the health districts, exercise specialists and/or nutritionists working at the HCs, and other HC staff members such as community health agents; in addition to older adults participants in the program.

### Reach

Described as the proportion of a target population that participates in an intervention (17). We assessed reach using quantitative data from recruitment, participation rate and representativeness of the population. To calculate participation rate, we used the number of participants attending the baseline assessment, divided by the number of individuals potentially eligible for the program. Additionally, barriers and facilitators of reach were assessed qualitatively. Interviews with HC personnel and city administrators sought to understand their perceptions about older adult participants, HCs, and program factors that could have influenced reach. Questions included: After the advertising of the project, how many older adults came in for the first meeting? How many older adults completed the entire baseline evaluation (e.g., physical test and answered the questionnaire)? How many older adults were engaged in the programs at a rate >75% of attendance? How many older adults dropped out the programs, meaning attending three or fewer sessions?

### Effectiveness

Measured at the individual level and reflective of the success of an intervention in improving health outcomes (17). To assess effectiveness, health measurements and questionnaires were collected from participants by trained researchers at two time points (baseline and immediately post-intervention). Evaluation included a social-demographic survey, quality of life assessment, anthropometric measurements and PA participation. Additionally, we conducted focus groups (FGs) to understand participants' perceptions. Questions included: What did you (participant) think of the program? What are your thoughts about program? How do you see your behavior (i.e., physical activity) after participating in the program? Do you think you are more physically active now? Has the program helped you to improve your lifestyle? How about your quality of life, did you perceive any changes?

#### Anthropometric Measurements

Weight was measured with the assistance of a medical weight scale. Height was measured with a stadiometer. Body mass index (BMI) scores were calculated and participants were classified as underweight (<18.5), normal weight (18.5–24.9) and overweight/obese (25.0 or more) (18).

#### Physical Activity

PA was assessed by GT3X and GT3X+ accelerometers and ActiLife® software was used to analyze the data. Each participant was instructed to use the accelerometer for 7 days in a row, removing it only to sleep, bathe or perform activities involving water. The device was attached to an elastic belt and fixed in the right side of the hip. Data were collected in a 30 Hz sample frequency and were analyzed using 60-s epochs. Periods with consecutive values of zero (with 2-min of spike tolerance) for 60 min or longer were interpreted as “accelerometer not worn” and excluded from the analysis (19). Physical activity data were included only when participants had accumulated a minimum of 10 h/day of recording, for at least 4 days, including one weekend day. The time spent in sedentary behavior (SED = 0–99 counts min^−1^) (20), in light physical activity (LPA = 100–2,689 counts min^−1^) and in moderate-to-vigorous physical activity (MVPA ≥ 2,690 counts min^−1^) (21) was calculated adjusting to the valid days and wear time. It was also analyzed the total time spent in SED bouts and the time spent in MVPA bouts by the sum of minutes spent in SED and MVPA, respectively, in periods lasting ≥10 min.

#### Quality of Life

Quality of life was evaluated by WHOQOL BREF and OLD questionnaire (22, 23). The WHOQOL-BREF instrument comprises of 26 items, which measure the following broad domains: physical health, psychological health, social relationships, and environment. The WHOQOL-OLD questionnaire comprises of 24 items which measure the following domains: sensory abilities; autonomy; past, present and future activities; social participation; death and dying; and intimacy. Each domain provides an individual score. Additionally, an overall score was calculated for each instrument (WHOQOL BREF and OLD).

### Adoption

Defined as the absolute number, proportion, and representativeness of settings and intervention agents who were willing to initiate a program (17). Adoption was evaluated using information from our database regarding the interest in adopting the programs, from regional district level through HC staff. We explored program adoption within hierarchical levels of the City Health Department using interviews and focus groups. Questions covered topics related to: What is your opinion about the program that was implemented in this health center in the last three months? How did the program change the health center routine? What do you think about the background and experience of the professionals in the health center and the degree to which they are confidently prepared to offer the program? Do the professionals in the health center have sufficient time available to deliver the program? What do you think was the most interesting aspect of the program? How do you see the benefits of the program? What do you think about the program cost?

### Implementation

At the setting level, implementation refers to staff fidelity to the various elements of an intervention's protocol. This includes consistency of delivery as intended and the time and cost of the intervention (17). We followed the implementation strategies (e.g., training) suggested by the program developers of ALED. ALED's check-list include 24 questions regarding implementation covering topics such as program fidelity, instructor knowledge, classroom, schedule, participants attention, attendance, among others. Programs implementation was assessed twice at each program site by two independent observers. In addition, interviews and focus groups with HCs personnel sought to understand their perceptions about the program implementation. Questions included: What was like to teach the program? What was most difficult and challenging? What was easy? What do you think motivates participation of older adults?

### Maintenance

Defined as the extent to which a program becomes institutionalized or part of routine organizational practices, maintenance also refers at the individual level to the long-term effects of a program on health outcomes (17). In our study, we focused on individual level maintenance. Similar data collection described in the effectiveness domain was carried out at 3 and 9 months after the intervention was concluded.

## Data Analysis

The demographic characteristics of the sample are presented in Table 2. Reach was analyzed using two-way ANOVA and chi-square tests to compare participants to those who declined to participate for each group and each location. Adoption rates were assessed by calculating the number of HCs that were approached and those that agreed to participate; as well as by assessing the percentage of professionals that agreed to deliver the programs. Although there are a multitude of definitions for implementation, for the purposes of the analyses, we focus on participation and adherence. As such, implementation rates were calculated by determining the proportion of participants completing at least 75% of program. Effectiveness and Maintenance were analyzed following procedures for clustered randomized control analysis. The generalized linear mixed model for repeated measures was used to conduct individual level outcome analysis and controlled the following characteristics as covariates (sex, age, schooling, sedentary time and PA in baseline). Additionally, variables with significant differences between groups (Table 2) were also included in the model as covariates. The Sidak *post-hoc* test was used to compare difference between groups at different assessment points. We used intention-to-treat (ITT) analysis to keep all participants with non-missing baseline outcome measurements.

**Table 2:** Participants' baseline characteristics by group.

**Variable**	**BCG**	**TEG**	**CG**	**Overall**	**Dif**
**Total number of participants** ***n*** **(%)**	**36 (31.6)**	**52 (45.6)**	**26 (22.8)**	**114 (100)**	
**Demographic variables**					
Age, mean (SD)	69.7 (6.9)	71.3 (7.3)	67.2 (5.8)	69.8 (7.0)	0.055^a^
Female, %	75.0	82.7	84.6	80.7	0.556^b^
Education level, %					0.139^b^
No studied	5.7	7.8	3.8	6.3	
Elementary school	51.4	68.6	76.9	65.2	
High school	20.0	13.7	19.2	17.0	
Higher	22.9	9.8	0.0	11.6	
Marital status, % married	65.7	52.9	46.2	55.4	0.189^b^
Monthly household income, %					0.267^b^
<2 salaries	27.8	34.6	50.0	36.0	
3–4 salaries	44.4	44.2	42.3	43.9	
More than 4 salaries	27.8	21.2	7.7	20.2	
Occupation, %					0.007^b^^**^
Retiree and/or pension	72.2	48.1	80.8	63.2	
Active works	27.8	51.7	19.2	36.8	
**Health/behavior variables**					
Health Status, %					0.669^b^
Good	52.8	44.2	53.8	49.1	
Fair	47.2	51.9	42.3	48.2	
Weak	0.0	3.8	3.8	2.6	
Disease, %					0.845^b^
Arthrosis	13.9	5.8	11.5	9.6	
Heart Disease	25.0	28.8	19.2	25.4	
High Blood Pressure	38.9	44.2	50.0	43.9	
BMI, Mean (SD)	27.4 (4.5)	28.4 (5.5)	27.8 (3.0)	27.9 (4.7)	0.609^a^
Weight, %					0.233^b^
Normal weight	47.2	34.6	53.8	43.0	
Overweight/obese	52.8	65.4	46.2	57.0	
PA Level (min/week) (SD)					
Sedentary time	498.5 (113.6)	529.8 (107.3)	522.8 (86.7)	518.3 (105.3)	0.391^a^
Light PA	315.5 (96.1)	301.6 (93.5)	292.8 (57.6)	304.1 (87.6)	0.600^a^
Moderate/vigorous PA	28.8 (24.2)	16.2 (17.9)	25.2 (26.1)	22.2 (22.6)	0.026^a^^*^

BCG, Behavior Change Group; TEG, Traditional Exercise Group; CG, Control Group.

* p <0.05,

** p <0.01.

a Anova One Way (factor group).

b Chi-square test.

The average of AFMV and occupation (ANOVA analysis) were larger in traditional groups than in the control groups (p <0.005).

*Florianopolis, Brazil, 2012 (n = 114)*.

Two Portuguese-speaking investigators transcribed the interviews and focus groups and checked them for accuracy. Transcript analysis was conducted in Portuguese. Deductive thematic analysis was conducted to identify themes/quotes within the RE-AIM model. A team of bicultural native Brazilian-Portuguese and English speakers translated the quotations through a translation/back-translation process to ensure semantic equivalence across languages (24, 25). This team reviewed each quotation for conceptual and normative equivalence (adapting and dropping items as needed to address cultural fit and social norms).

## Results

Table 2 presents participant (*n* = 114) characteristics at baseline. Findings show a similarity among demographic and health variables across the three groups (BCG, TEG, CG). Average age was 69.8 years, the majority were women (80.7%) with high school education or less (88.5%). Approximately, 57% were overweight or obese with an average BMI of 27.9 kg/m^2^. There was a high prevalence of chronic diseases with 43.9% reporting high blood pressure and 25.4% heart disease. The only significant difference among groups at baseline was regarding occupation and MVPA, where participants in the traditional group were more likely to be working and were found to be engaged in less moderate/vigorous PA.

### Reach

Results are shown in Figure 1. Among the 4,071 older adults across the six HCs, 985 individuals (24.2%) were considered eligible for the study. A total of 114 individuals completed the baseline assessment, representing a reach of 11.5%. Overall 49% of participants attended at least 75% of all sessions with disengagement occurring mostly in the first three weeks of the study (42%). TEG showed highest reach (15.3%) followed by BCG (12.1%) and CG (8.1%), respectively. Several barriers affecting the program's reach were identified. System's level instabilities that often lead to inconsistent support for health services was a common barrier mentioned in interviews. This issue was captured in this quote from a community health worker recruiter for the study: “*We were having a lot of turbulence in the unit [HC], we almost could not get involved. It was soon after that we were without the doctor. And, after then, the doctor went on vacation, so for me it was a very hard time to work.”* (CCS BL). Another barrier deals with older adults' resistance to change. A community health worker described “*Older adults struggle with new things…they fear if we invite them for something new, they are scared…*” (ACS ST). A final barrier for recruitment was lack of staff familiarity with behavior change programs. Innovative and new to most staff in the HCs, the program was relatively difficult to explain and to understand. A community health worker described this concern when saying “*Very often the team could not understand the concept of the program [referring to the behavior change program]*” (PEFI).

On the other hand, several facilitators for reach were identified. Doctors and other health professionals encouraging patient participation was viewed as favorable in interviews. One community health worker noted “*The involvement of the health team was very important*” (PNS CAN). In addition, the program materials were perceived to be attractive by participants. An older adult participant said “*It was very good [referring to the program recruitment material], because for me it was essential, you know why I do not live without it [laughs] that motivates you there*…” (BP3). Distribution of flyers and personal invitations were effective ways to reach participants. This was illustrated by a community health worker who stated “*We have a definite demand of people attending the center, we are capable of attending a defined number, but by word of mouth the information gets through and we will always have someone else attending, so I believe there would be greater attendance, a bigger group*” (PNS ST).

### Effectiveness and Maintenance

Table 3 reports the statistical summary of outcomes for physical activity behavior, BMI, quality of life over a 12-month period comparing the three groups (BCG, TEG, and CG). There was a reduction in sedentary behavior after the intervention for BCG (*P* < 0.05). This result was maintained at 3- and 9-month post-intervention evaluations (*P* < 0.01). Participants in the BCG increased their MVPA behavior at post program assessment, and maintained their physical activity participation levels at 3-month post-intervention. Neither the TEG, nor the CG, increased their MVPA, experiencing significant declines in MVPA over time (*P* < 0.05). No difference was observed among groups and no time effect was observed in BMI. Participants in the BCG showed significant improvements in quality of life (WHOQOL-*Brief*) at 9-month post-intervention relative to baseline (*P* < 0.031).

**Table 3:** Estimated mean change in physical activity behavior.

**Outcome variable**	**Mean (SE)**			***P*****-Value**		
	**BCG**	**TEG**	**CG**	**BCG vs. CG**	**TEG vs. CG**	**BCG vs. TEG**
**INTENT-TO-TREAT**						
**Change in sedentary behavior, min/day**						
At 3 months	−14.3 (56.3)^*^	−4.1 (62.2)	−25.6 (77.9)^**^	0.999	0.634	0.987
At 6 months	−16.6 (46.0)^**^	16.4 (97.9)^*^	−0.6 (80.1)	0.954	0.103	0.010^*^
At 12 months	−10.9 (59.9)^**^	4.2 (78.6)	−26.7 (68.3)^**^	0.976	0.216	0.520
**Change in light PA, min/day**						
At 3 months	−1.9 (30.3)	3.2 (57.9)	21.1 (40.5)	0.334	0.453	0.987
At 6 months	19.1 (40.1)	−4.7 (51.3)	41.4 (70.5)	0.278	0.007^*^	0.435
At 12 months	2.4 (42.6)	−15.0 (52.6)	10.1 (56.2)	0.855	0.068	0.588
**Change in MVPA, min/day**						
At 3 months	4.8 (14.1)^*^	3.8 (23.9)	−1.2 (21.0)	0.469	0.555	0.988
At 6 months	−0.2 (17.8)	−2.7 (10.5)	−5.2 (15.1)^*^	0.357	0.789	0.646
At 12 months	0.6 (18.0)	−3.6 (9.8)^*^	−4.9 (20.3)^*^	0.219	0.965	0.168
**Change in BMI, kg/m**^**2**^						
At 3 months	−0.1 (0.3)	−0.1 (0.7)	0.2 (0.5)	0.644	0.419	0.765
At 6 months	0.1 (0.5)	−0.1 (1.2)	0.1 (0.7)	0.765	0.536	0.234
At 12 months	0.1 (0.5)	−0.1 (0.8)	0.3 (1.1)	0.578	0.021^*^	0.346
**Change in WHOQOL brief**						
At 3 months	2.2 (5.4)	2.4 (6.9)	1.5 (8.9)	0.467	0.876	0.865
At 6 months	1.3 (7.9)	1.4 (7.2)	0.8 (9.6)	0.534	0.423	0.234
At 12 months	3.0 (7.8)^*^	1.0 (6.6)	1.7 (10.3)	0.986	0.456	0.765
**Change in WHOQOL old**						
At 3 months	2.4 (7.3)	1.5 (6.9)	2.7 (10.1)	0.943	0.965	0.942
At 6 months	2.4 (7.3)	1.4 (6.3)	4.4 (10.8)	0.673	0.897	0.761
At 12 months	2.3 (7.8)	−0.1 (7.3)	2.9 (11.0)	0.996	0.767	0.611
**COMPLETE CASES**						
**Change in sedentary behavior, min/day**						
At 3 months	−26.0 (75.2)^*^	−5.4 (78.3)	−29.3 (82.6)^*^	0.371	0.918	0.010^*^
At 6 months	−27.2 (57.4)^**^	62.6 (130.9)	4.9 (74.9)	0.494	0.103	0.020^*^
At 12 months	−16.7 (98.5)^**^	−4.3 (83.8)	−21.6 (64.7)^*^	0.567	0.308	0.057
**Change in Light PA, min/day**						
At 3 months	−26.0 (17.2)	−5.4 (13.6)	−29.3 (17.6)	0.999	0.619	0.748
At 6 months	−27.2 (14.3)	62.6 (23.1)	4.9 (16.3)	0.375	0.256	0.027^*^
At 12 months	−16.7 (31.1)	−4.3 (15.8)	−21.6 (15.7)	0.967	0.970	0.698
**Change in MVPA, min/day**						
At 3 months	8.7 (4.1)^*^	1.5 (2.5)	−1.3 (4.5)	0.010^*^	0.762	0.023^*^
At 6 months	−2.8 (6.1)	−4.4 (2.3)	−3.9 (3.1)	0.083	0.245	0.087
At 12 months	2.0 (8.4)^*^	−6.4 (2.3)^*^	−2.1 (5.0)	0.069	0.546	0.013^*^
**Change in BMI, kg/m**^**2**^						
At 3 months	−0.5 (0.1)	−0.1 (0.1)	0.2 (0.1)	0.883	0.997	0.998
At 6 months	0.1 (0.2)	−0.2 (0.2)	0.1 (0.2)	0.996	0.876	0.965
At 12 months	0.1 (0.2)	−0.2 (0.1)	0.5 (0.3)	0.679	0.786	0.987
**Change in WHOQOL brief**						
At 3 months	4.4 (1.6)	4.1 (1.5)	1.7 (1.9)	0,678	0.564	0.998
At 6 months	2.9 (2.7)	3.1 (1.4)	1.6 (1.9)	0.996	0.876	0.896
At 12 months	3.7 (2.2)^*^	2.1 (1.5)	2.2 (2.5)	0.987	0.787	0.976
**Change in WHOQOL old**						
At 3 months	4.8 (2.3)	2.5 (1.6)	3.0 (2.1)	0.987	0.645	0.654
At 6 months	5.0 (2.4)	2.1 (1.4)	4.9 (2.3)	0.675	0.786	0.876
At 12 months	2.3 (3.4)	−0.7 (1.9)	1.9 (2.8)	0.986	0.876	0.054

BCG, Behavior Change Group; TEG, Traditional Exercise Group; CG, Control Group.

* p < 0.05,

** p < 0.01 Adjusted.

*BMI, quality of life over a 12-month period. Florianópolis, Brazil, 2012*.

Analysis of the interviews and focus groups suggest that many of the participants perceived positive changes in themselves as a result of participating in the study. One participant in the BCG stated “*I needed to be physically active, I thought that I was limited by my illness, but I want to reach program targets and, I started walking more*” (12 BLP). Participants also described their appreciation with regard to the program effectiveness. “*Oh, I learned a lot with the program, mainly the walking activities, it was also good to know the number of steps we take, this device (pedometer) is great*” (P1).

The satisfaction with results achieved was noted by a participant with cardiovascular disease and obesity, as follows: “*(…) I started to take the program seriously (…) the activities are important, today I feel satisfied with my weight loss, some people lost five, ten and after until twenty kilos (refer the lose weight), a little every day, I am very satisfied* (14 BLP)”.

### Adoption

The program was approved by the director of City Health Department and by the management of NASF (Family Health Support Centers). Three of the five health districts declined to participate due to prior commitments or no interest in the study. Of the 18 health centers located in the two participating HDs, six agreed to participate in the study. Reasons for non-participation included health educators declining to participate for work or health reasons (*n* = 5), HCs with insufficient physical space to offer the programs (*n* = 5) and, HC professionals simply declining to participate (Figure 1).

These following quotations illustrate the heavy workload reported by the health center teams: “*The staff in this center is already over committed, they cannot handle extra activities*” (ACS BL); “*Everybody is busy an d overworked*” (ACS ST); “*I cannot let the staff dedicate eight hours per week to the program during the training period when the program is focused on only twenty or thirty people and our demand is much greater than that and they must take care of others areas*” (G2).

### Implementation

Two researchers with expertise in RE-AIM and the ALED program assessed program implementation twice in each study site. The evaluation for all items under analysis achieved an average of 98% fidelity. The estimated cost per participant for 3 months in the BCG program was R$ 65 (about U$ 30) and the TEG was R$ 50 per month (about U$ 23). Overall, 47% of TEG participants attended at least 75% of the sessions compared to 27.3% of their BCG counterparts. When managers were questioned regarding the cost of the programs, they found it to be expensive, as shown by the following quotes: “…*it is expensive, considering what is financially available from the government to each person, what is given to the cities, the fixed minimum wage, which is very low, the program is expensive…*” (G1). “*We would have to quit a program like “Floripa Ativa” or the walking group to include this new program…*” (PEF3).

## Discussion

This study evaluated the role of public health centers (HC) in the promotion of physical activity programs among older Brazilians, and compared the impact of two program strategies, behavioral change and traditional exercise. The RE-AIM framework was used to ground the evaluation of our programs. This framework has been previously used to evaluate programs offered in real-world settings (16, 26, 27). The use of RE-AIM in this study represents an innovation in public health program evaluation in Brazil. As stated by Glasgow, the knowledge generated by the RE-AIM goes beyond literal translational research, and supports program adaptations to various cultures and populations (11).

Our analysis investigated the reach, efficacy, adoption, implementation, and maintenance of RE-AIM components by collecting qualitative and quantitative data from program participants and partners at the organizational level. Our findings reveal both strengths and areas for improvement of the program strategies, and identified important factors associated with the utilization of public health centers in such initiatives.

Results revealed a limited *reach* of the programs offered at public health centers, with participation levels of about 12% of the older adult population. This finding suggests that future studies should seek to understand better older adults' resistance to change, and to develop culturally-sensitive strategies to overcome these barriers to reach. A systematic review published by Franco et al. (28) on barriers and facilitators to physical participation among activity older adults revealed that some individuals believe that physical activity is unnecessary or even potentially harmful. Others recognize the benefits of physical activity, but report a range of barriers to physical activity participation. The authors describe the importance of raising awareness of the benefits, educating about incorrect perceptions regarding the risks of physical activity, and improving environmental and financial access to physical activity opportunities. While building on the status and respect paid to leaders in public health centers to promote reach, such as doctors encouraging patient participation and personal invitations from community health workers.

The reach *and representativeness* of our program could be improved with a stronger level of support from administrators responsible for the planning and scheduling of health services, so that the programs become less susceptible to systems-level instabilities. *While program participants presented very similar demographic characteristics of non-participants, the program was only offered to 2 participating health districts, so with three health district coordinators declining participation, it led to 30 HCs never having the opportunity to hear about the program. The* complex operational problems in SUS/HCs have been previously documented (29) and the solutions require multi-faceted public health actions. *Reach and representativeness could be improved by greater buy-in from operational leaders and changes throughout the system to increase the number of health districts offering the program*.

Results of the program *effectiveness* and *maintenance* assessments reveal trends favoring behavior change strategies over traditional exercise classes. Behavior change programs were more successful in decreasing sedentary time and increasing moderate-to-vigorous physical activity, as well as improving participants' quality of life. Overall, participants perceived positive changes in themselves as a result of participating in the programs. They reported satisfaction with program results, improved awareness of the importance in being active, and were able to find opportunities to be active in daily routines. The effectiveness of the behavior change program (ALED) is consistent with the study by Baruth et al. (30) who reported clinically meaningful improvements in performance-based measures of physical functioning. Dunn et al. (10) also noted that a behaviorally based lifestyle physical activity intervention can significantly increase physical activity levels. They concluded that health care professionals who counsel their patients about physical activity can provide options beyond traditional fitness center–based recommendations.

Our results regarding program maintenance were promising, as many improvements post-intervention persisted in follow up. This is an important finding as maintenance has often been an overlooked dimension of RE-AIM and a common limitation of programs' evaluation. Galaviz et al. (24) describe important methodological limitations to the assessment of maintenance, such as the low participation of subjects in follow-up measurements.

We had an overall 14% rate of program *adoption* by public health centers. This is somewhat disappointing, considering the efforts by program developers in building partnerships with stakeholders across all hierarchical levels within the local health system. Our findings suggest that several organizational level factors hindered greater adoption of the programs, including heavy workload of health professionals delivering the program and limited physical space for program delivery. Without question, adoption depends on the commitment of local health teams. Schrader et al. (31) raised concerns about workload of health teams in Brazil that prevent them from engaging in activities beyond the typical assignment. King (32) underscored the importance of health care providers promoting physical activity interventions for older adults. However, the culture of managing chronic diseases at health care centers in Brazil is often associated with more traditional medical or pharmaceutical approaches including drug prescription (33). A deconstruction of these health care settings is necessary to engage patients and health professionals in health promotion and improve adoption of such programs.

The fidelity of program delivery was high and indicates that both programs are culturally- appropriate for the Brazilian context and feasible to be *implemented* by local health educators. Results showed that training of staff members was adequate and effective. As Brazil is lacking evidence-based behavior change programs for older adults, disseminating US-developed health programs, such as ALED, seems a viable option. Liu et al. have supported similar efforts in China (34). Additional implementation factors examined in this study included the cost of program implementation, which placed traditional exercise classes at an advantage over behavior change programs. However, regardless of program type, administrators of public health centers reacted negatively to any additional cost that the programs added to their budgets or for participants. This is clearly an implementation issue that requires further attention. Finally, both intervention groups showed relatively high disengagement rates (BCG 50% vs. TEG 37%) with individuals in the BCG presenting lower rates of overall attendance (27 vs. 47%). Nevertheless, our effectiveness and maintenance results showing greater sedentary behavior, MVPA, and quality of life improvements in BCG, suggests that the behavioral modification strategies (tailored goal setting, self-monitoring, action planning, feedback) presented at the on-site meetings and throughout the written materials may have had the desired impact in helping individuals engage in healthful behaviors even when not attending sessions. These results are not unlike current literature on lifestyle modification interventions where a recent review on factor associated with adherence to lifestyle modification programs for weight management found adherence rates to vary from 20 to 80% of participants attending three or less sessions (35). Clearly, attendance continues to be a challenge for these types of programs and future studies should continue to explore strategies to improve overall attendance rates. In particular, the use of automated tracking tools (i.e., Fitbit, pedometers) have shown some early promise (36). Translating research into policy and practice is both a need and a challenge. As described by Oelke et al. (37) there are many barriers to disseminating and using research results in the Brazilian health care setting, including little involvement of key stakeholders and lack of partnerships between researchers and knowledge-users in research process, low research budgets and limited support by funding agency policies.

## Conclusion

While participant attendance remains a challenge, this study supports the potential for dissemination of behavior change and traditional exercise programs to older adults through public health centers in Brazil. Our study advances the health literature by examining individual- and system-level factors associated with the promotion of physical activity in this aging society.

## Data Availability Statement

The datasets generated for this study are available on request to the corresponding author.

## Ethics Statement

The studies involving human participants were reviewed and approved by the ethics committee of the Federal University of Santa Catarina (CONEP n. 480560 and CEPSH n. 2387/2010). The patients/participants provided their written informed consent to participate in this study.

## Author Contributions

TB designed the research. FA guided on the design and statistical analyses. CR analyzed the data. LK, FB, WC-Z, and AS contributed to data collection and interpretation of findings. TB, CR, LK, FA, FB, WC-Z, and AS wrote the manuscript and TB had primary responsibility for final content. All authors contributed to the manuscript editing, read, and approved the final manuscript.

### Conflict of Interest

The authors declare that the research was conducted in the absence of any commercial or financial relationships that could be construed as a potential conflict of interest.

## Abbreviations

PA: Physical activity (PA)
HCs: Public health centers
SUS: Unified health system
TEG: Traditional exercise group
BCG: Behavioral change group
CG: Control group
FGs: Focal groups
BMI: Body mass index
SED: Sedentary behavior
LPA: Light physical activity
MVPA: Moderate-to-vigorous physical activity.
